# Supplementary material for: Molecular Differences Based on Erythrocyte Fatty Acid Profile to Personalize Dietary Strategies between Adults and Children with Obesity
Source: Metabolites. 2021 Jan 8;11(1):43. doi: 10.3390/metabo11010043 (PMC7827034; doi:10.3390/metabo11010043)
Supplement: Supplementary file 1 [file metabolites-11-00043-s001.zip › Manuscript.docx]

**Molecular differences based on erythrocyte fatty acid profile to personalize dietary strategies between adults and children with obesity.**

**Iker Jauregibeitia^1^, Kevin Portune^1^, Sonia Gaztambide^2^, Itxaso Rica^2^, Itziar Tueros^1^, Olaia Velasco^2,^ Gema Grau^2^, Alicia Martín^2^, Luis Castaño^2^, Anna Vita Larocca^3^, Federica Di Nolfo^3^, Carla Ferreri^4*^  and Sara Arranz^1*^**

^1^AZTI, Food Research, Basque Research and Technology Alliance (BRTA). Parque Tecnológico de Bizkaia, Astondo Bidea, Edificio 609, 48160 Derio - Bizkaia.

^2^Biocruces Bizkaia Health Research Institute, Cruces University Hospital, CIBERDEM/CIBERER, UPV/EHU, Endo-ERN, 48903 Barakaldo, Spain.

^3^Lipidomic Laboratory, Lipinutragen srl, Via di Corticella 181/4, 40128 Bologna, Italy

^4^Consiglio Nazionale delle Ricerche, ISOF, Via Piero Gobetti 101, 40129 Bologna, Italy.

*Correspondence: carla.ferreri@isof.cnr.it (C.F.); sarranz@azti.es (S.A.); Tel.: +39‐051‐6398289 (C.F.);

+34‐671750399 (S.A.); Fax: +39‐051‐6398350 (C.F.); +34‐946‐572‐555 (S.A.)

**Abstract**

As the obesity epidemic continues to grow inexorably worldwide, the need to develop effective strategies to prevent and control obesity seem crucial. The use of molecular tools can be useful to characterize different obesity phenotypes in order to provide more precise nutritional recommendations. The aim of this study was to determine the fatty acid (FA) profile of red blood cell (RBC) membranes, together with the evaluation of their dietary intake and biochemical parameters, of children and adults with obesity. An observational study was carried out on 196 children (113 with normal weight and 83 with obesity) and 91 adults (30 with normal weight and 61with obesity). Mature RBC membrane phospholipids were analysed for FA composition by gas chromatography-mass spectrometry (GC-MS). Dietary habits were evaluated using validated food frequency questionnaires (FFQ). Children with obesity presented higher levels of ω-6 polyunsaturated FAs (mainly linoleic acid, p=0.01) and lower values of ω-3 FAs (mainly DHA, p<0.001) comparing with adults. Regarding blood biochemical parameters, children with obesity presented lower levels of glucose, LDL cholesterol and alanine aminotransferase compared with adults with obesity. These differences should be considered to provide specific nutritional recommendations for different age groups, based on an adequate fat intake.

**Keywords:** lipid metabolism, mature erythrocyte, obesity, precision nutrition.

1. **Introduction**

In the last decades, unhealthy dietary patterns are increasing and affecting the prevalence of noncommunicable diseases in the world. According to the World Health Organization (WHO), worldwide obesity has nearly tripled since 1975, indicating that, in 2016, 39% of adults aged 18 years and over, were overweight and 13% suffered from obesity([1](#_ENREF_1)). In addition to affecting the adult population, obesity is becoming a rising problem affecting children and adolescents as well. As the WHO states, more than 340 million children and adolescents, around one in three from 5 to 19 years, were overweight or obese in 2016 and 38 million children under the age of 5 were overweight or obese in 2019 ([1](#_ENREF_1)).

Obesity prevention and treatment strategies include lifestyle and behavioural interventions, focused on changes in diet and physical activity. Low-fat diets, to reduce calory intake, have been the most recommended strategy for people with obesity in the past decades ([2](#_ENREF_2)). Moreover, there is not enough evidence from randomized control trials supporting beneficial effects of low-fat diets over other dietary interventions for long-term weight loss.([2](#_ENREF_2)) Besides, recent scientific evidence showed that low fat diets reduced LDL and HDL cholesterol and increase triglycerides. Further, the replacement of saturated fatty acids (SFA) with monounsaturated fatty acids (MUFA) has been proposed as an appropriate strategy to reduce obesity, since substituting SFA with MUFAs raises HDL-cholesterol levels, improves insulin sensitivity and lowers LDL-cholesterol levels([3-5](#_ENREF_3)). Other dietary plans have been proposed including low-carbohydrate diets, high-protein diets, very low-caloric diets with meal replacements, Mediterranean diet, and diets with intermittent energy restrictions, evidencing that a successful diet must be healthy, balanced and without nutritional deficiencies. In any case, most of them include general dietary recommendations rather than specific dietary plans based on individual metabolism([6](#_ENREF_6)).

However, obesity prevalence in both child and adult populations continues increasing worldwide, suggesting that, personalized intervention strategies could provide precise nutritional guidance and contribute to successful long term interventions([7](#_ENREF_7)). Even though dietary guidelines for macronutrients intake in adults and children are established([8](#_ENREF_8), [9](#_ENREF_9)), according to the different requirements of both population groups, specially from energy intake, interventions to control obesity in children and adults are not specific nor differentiated, regarding the intake of food groups or specific nutrients. For that reason, the optimal macronutrient distribution of the diet to improve weight status is unclear([10](#_ENREF_10)). The use of molecular tools (metabolomic, nutrigenetic, metagenomic, etc) can provide new scientific evidence related to the characterization of different obesity phenotypes together with the impact of diet on metabolism ([11](#_ENREF_11)). This can be useful to personalize therapy and contribute to provide more precise nutritional recommendations, mainly for an adequate fat intake for different age groups and health conditions([12-14](#_ENREF_12)).

The use of mature erythrocyte membrane as a representative site for all other body tissues in FA profiling, is an established protocol for membrane-based molecular diagnostics ([15-17](#_ENREF_15)). The measure of the lipid profile at the cellular level, precisely at the membrane phospholipid level, provides not only information related to the nutritional status of an individual, but also information related to FA metabolism that is involved in the formation of the most important lipid building blocks for cell life, which are the phospholipids. This approach has a profound diagnostic meaning, not only from the biochemical point of view related to the lipid pathways, but also from the biophysical and biological consequences, since the balance reached by the FA components of the membrane phospholipids must respect the tissue type and, ultimately, satisfy the homeostatic requirement for the optimal cell functioning([18](#_ENREF_18)) .

The aim of this study was to evaluate lipid profile differences in mature RBC membranes between children and adult with obesity, in relation to their nutrient intake. Defining these differences in RBC FA profiles, related to individual molecular and nutritional status, will allow the design of differentiated nutrition strategies for children and adults with obesity, giving relevance to the functional roles of the different fatty acid residues in lipids.

1. **Materials and methods**
   1. **Subjects and study design**

An observational, case-control and retrospective study was conducted on 83 children with obesity (26 boys and 57 girls) between 6 to 16 years old and a group of 61 adults with obesity (19 males and 42 females) between 19 to 68 years old, recruited from pediatric endocrinology and the endocrinology department at the Hospital Universitario Cruces (Barakaldo. Spain). Control subjects, consisting of 113 normoweight children and 30 normoweight adults, were also recruited from the same centers as patients with obesity. Children were classified according to body mass index (BMI), using age and sex-specific pediatric z-scores from Orbegozo tables ([19](#_ENREF_19)). The BMI was taken as a reference to define the different categories, defining normal weight when the standard deviation (SD) of BMI was -1 <SD ≤ +1, overweight when+1<SD ≤ +2, and obesity when SD> +2. For adults, BMI>30 Kg/m^2^ was taken as reference to classify obesity and 18.5<BMI<25 Kg/m^2^ for the normoweight group.

Subjects were excluded if they presented any kind of acute or chronic diseases, were taking medications, had any presence of metabolic syndrome symptoms or obesity associated to any type of pathology. A physical examination was performed by an endocrinologist.

The study protocol was approved by the Euskadi Clinical Research Ethics Committee (permission number PI2016181) and carried out according to the Declaration of Helsinki Good Clinical Practice guidelines. Subjects under study were included after acceptance (by the parents in the case of the pediatric population) to participate in the study and signing of informed consent. In the case of children between 12-16 years of age the informed consent was also signed by themselves according to the Euskadi Ethical Committee and sample biobank laws (Organic Law 3/2018, of December 5, on Protection of Personal Data and guarantee of digital rights; Law 14/2007 on Biomedical Research and RD 1716/2011 of Biobanks).

***2.2 Anthropometric measures***

Body weight (kg) and height (cm) were measured by standardized methods([20](#_ENREF_20)). Body mass index (BMI) was calculated as weight (kg) divided by the square of the height (m^2^). Anthropometric parameters, as well as blood sampling were all conducted by pediatricians and doctors during the participant’s visit to the Hospital Universitario Cruces/IIS Biocruces Bizkaia.

***2.3 Nutrient Intakes***

During the participant´s visit with the endocrinologist, the doctor interviewed the participants and collected personal data, including family medical history and information on the history of medication usage. Estimations of food consumption, including dietary diversity and variety, were measured using a quantitative food frequency questionnaire (FFQ) on-line completed by the parents of the children, except in those cases of adolescents, which were encouraged to complete it themselves, or by each adult volunteer. For our study, an adapted FFQ was used, which was previously validated with the portion sizes and food groups for the Spanish juvenile population and for adults ([21](#_ENREF_21), [22](#_ENREF_22)). These questionnaires were then analyzed using the DIAL® software (UCM & Alce Ingeniería S.A. Madrid. Spain) (V 3.4.0.10) to translate the intake of specific foods into their corresponding energy and nutrient values.

***2.4 Red Blood Cell (RBC) Membrane Fatty Acid Analysis***

The fatty acid composition of mature RBC membrane phospholipids was obtained from blood samples (approximately 2 mL) collected in vacutainer tubes containing ethylenediaminetetraacetic acid (EDTA). Samples were shipped to the Lipidomic Laboratory approved for the method by the UNI CEI EN ISO/EIC 17025:2018 (#1836L belonging to the company Lipinutragen, Bologna, Italy) and upon arrival underwent the certified procedure MEM_LIP_1 according to the quality control guidelines. At first, the absence of hemolysis was checked upon arrival. From the blood, the protocol consists of selection of mature RBCs by a robotic platform, as reported previously([17](#_ENREF_17), [23-25](#_ENREF_23)), followed by lipid extraction and lipid transesterification to fatty acid methyl esters (FAMEs). Briefly, the whole blood in EDTA was centrifuged (4000 rpm for 5 min at 4ºC), and the mature cell fraction was isolated by the robotic platform, based on the higher density of the aged cells, ([26](#_ENREF_26)) and checked by the use of cell counter (Scepter 2.0, EMD Millipore, Darmstadt, Germany). The automation included cell lysis, isolation of the membrane pellets, phospholipid extraction from pellets using the Bligh and Dyer method ([27](#_ENREF_27)), transesterification to FAMEs by treatment with a potassium hydroxide (KOH)/methyl alcohol (MeOH) solution (0.5 mol/L) for 10 min at room temperature, and extraction using hexane (2 mL). The final FAME mixtures were analyzed using capillary column gas chromatography (GC). GC analysis was run on the Agilent 6850 Network GC System, equipped with a fused silica capillary column Agilent DB23 (60 m x 0.25 mm x 0.25 μm) and a flame ionization detector. Optimal separation of all fatty acids and their geometrical and positional isomers was achieved. Identification and quantification of each fatty acid was made by calibrated procedures that are part of the MEM_LIP_1 method. Commercially available standards and a library of trans isomers of MUFAs and polyunsaturated fatty acids (PUFA) were used as standards. The amount of each FA was calculated as a quantitative percentage of the total FA content (relative quantitative %), as described in section 2.5, being more than 97% of the GC peaks recognized with appropriate standards.

***2.5 Red Blood Cell Membrane Fatty Acid Cluster***

12 FAs were selected as a representative cluster of the dominant glycerophospholipids present in the RBC membrane, as well as three FA families (SFA, MUFA and PUFA): for SFAs, palmitic acid (C16:0) and stearic acid (C18:0); for MUFAs, palmitoleic acid (C16:1;9c), oleic acid (C18:1; 9c), cis-vaccenic acid (C18:1; 11c); for ω-3 PUFAs, eicosapentaenoic acid (EPA) (C20:5), docosahexaenoic acid (DHA) (C22:6); for ω-6 PUFAs, linoleic acid (LA) (C18:2), dihomo-gamma-linolenic acid (DGLA) (C20:3) and arachidonic acid (AA) (C20:4); for geometrical trans fatty acids (TFA): elaidic acid (C18:1 9t) and mono-trans arachidonic acid isomers (monotrans-C20:4; ω-6 recognized by standard references as previously described by Ferreri et al([28](#_ENREF_28)). Considering these fatty acids, different indexes previously reported in the literature ([25](#_ENREF_25)) were calculated: (%SFA/%MUFA) index related with membrane rigidity; Omega-3 index (DHA + EPA); Inflammatory risk index (% ω-6)/(% ω-3); PUFA balance [(%EPA + %DHA)/total PUFA x 100]; Free radical stress index (sum of trans-18:1 + Σ monotrans 20:4 isomers); Unsaturation Index (UI) [(%MUFA) + (%LA/2) + (%DGLA/3) + (%AA/4) + (% EPA/5) + (%DHA/6)]; Peroxidation Index (PI) [(%MUFA/0.025) + (%LA) + (%DGLA/2) + (%AA/4) + (% EPA/6) + (%DHA/8)]; De Novo Lipogenesis index (DNL) [(%Palmitic acid)/(%LA)]([29](#_ENREF_29)).

Additionally, the enzymatic indexes of elongase and desaturase enzymes, the two classes of enzymes of the MUFA and PUFA biosynthetic pathways, were inferred by calculating the product/precursor ratio of the FAs involved in these reactions.

***2.6 Biochemical parameters***

Blood biochemical parameters were measured with standard laboratory assays after collecting venous blood samples performed in the morning in fasting state from a subgroup of the studied population (69 children vs. 44 adults with obesity and 34 paediatric vs. 30 adults with normal weight). Plasma concentrations of glucose, serum concentrations of total cholesterol (TC), high-density lipoprotein cholesterol (HDL-C), low-density lipoprotein cholesterol (LDL-C), triglycerides (TG), aspartate aminotransferase (AST), alanine aminotransferase (ALT), uric acid and bilirubin were measured.

***2.7 Statistical Analysis***

Differences between groups for the nutrient intake and biochemical values were determined by using the Mann-Whitney U test for data that was not normally distributed and the t-student test for normally distributed variables. Normal data distribution was assessed by Shapiro-Wilk's test and Kolmogorov-Smirnov test.

An ANCOVA was run to determine the differences between RBC membrane fatty acids from children and adults with obesity, and also for normoweight children and normoweight adults, after controlling for variables selected as potential confounders, such as gender, BMI and dietary intake ([30](#_ENREF_30)). Post hoc analysis was performed with a Bonferroni adjustment. First, a Principal Component Analysis (PCA) was run on 15 dietary nutrient intake variables (individual FAs, families (SFA, MUFA, and PUFA), total lipids, carbohydrates and proteins) obtained with the DIAL software (v3.4.0.10, Department of Nutrition (UCM) & Alce Ingeniería, S.L., Madrid, Spain) after transforming the information about food items from FFQ questionnaires into micro and macronutrient values, in order to reduce and simplify the dimension of these variables and use the generated factors as diet covariates ([25](#_ENREF_25)). The Kaiser-Meyer-Olkin (KMO) and Bartlett’s test of sphericity were used to verify the sampling adequacy for the analysis. PCA revealed four components that had eigenvalues greater than one and which explained 82.55% of the total variance. These components were included in the ANCOVA analysis as diet covariates. The level of signiﬁcance was set at *p* < 0.05.

Correlations between the RBC membrane FA profile and dietary intake and FA profile and biochemical values were performed using Spearman’s rank-order correlation coefficients (ρ) and p-values were adjusted with the false discovery rate method for multiple comparisons. Correlation plots were visualized using the R heatmap.2() function. All other statistical analyses were performed using SPSS (IBM Corp. v24.0, Armonk, NY, USA).

1. **Results**
   1. ***Red blood cell membrane fatty acids profile***

The pediatric group with obesity showed lower levels of palmitic acid and cis-vaccenic acid (*p* =0.01 and *p*=0.05 respectively) compared to the adult group with obesity (Table 1). Linoleic acid, DGLA and total ω-6 FA levels in pediatric group with obesity were higher compared with the adult group with obesity (*p* =0.01, *p* =0.04 and *p* <0.01 respectively). DHA and total ω-3 FA levels were lower for the pediatric group with obesity (*p*<0.01 and *p*<0.01 respectively) and hence, ω-6/ω-3 ratio was higher (*p*<0.01). Regarding other indexes, PUFA balance, PI and UI were lower for the pediatric group with obesity (*p*<0.01, *p*=0.01 and *p*= 0.04 respectively).

After observing these differences, a sample of normoweight adults (30) and children (113) were analyzed in order to determine if these differences observed between the groups with obesity were due only to age differences or whether they could be attributed to metabolic differences. Similar patterns for LA, DGLA, DHA, total ω-3 FA levels, total ω-6 FA levels and ω-6/ω-3 ratios were observed in the normoweight populations, but no differences for palmitic acid, cis-vaccenic acid or Unsaturation Index were observed when comparing adult and child populations with normal weight. The DNL index (19), which is the ratio between C16:0/C18:2 ω-6 fatty acids, that correlates directly with the liver fat content, appears in higher levels for adults with normal weight and obesity when compared with the respective child populations (p<0.01 for both).

**Table 1.** Red blood cell **(**RBC) membrane fatty acid profile.

|  | **Group with obesity** | | | |  | **Group with normal weight** | | | | |
| --- | --- | --- | --- | --- | --- | --- | --- | --- | --- | --- |
|  | **Pediatric n=83** | | **Adult n=61** | | **Ancova** | **Pediatric n=113** | | **Adult n=30** | | **Ancova** |
| **Fatty Acid (%)** | Mean | SE | Mean | SE | *p*^*^ | Mean | SE | Mean | SE | *p*^*^ |
| Palmitic acid (C16:0) | 22.31 | 0.16 | 23.22 | 0.22 | **0.01** | 22.51 | 0.10 | 22.72 | 0.23 | 0.43 |
| Stearic acid (C18:0) | 18.22 | 0.18 | 17.54 | 0.24 | 0.06 | 17.68 | 0.10 | 17.66 | 0.23 | 0.94 |
| TOTAL SFA | 40.55 | 0.16 | 40.78 | 0.21 | 0.48 | 40.21 | 0.10 | 40.22 | 0.23 | 0.97 |
| Palmitoleic acid (C16:1) | 0.46 | 0.02 | 0.37 | 0.03 | 0.08 | 0.41 | 0.02 | 0.48 | 0.03 | 0.08 |
| Oleic acid (9c C18:1) | 16.55 | 0.20 | 17.08 | 0.27 | 0.20 | 17.46 | 0.12 | 17.79 | 0.27 | 0.29 |
| cis-Vaccenic acid (11c C18:1) | 1.17 | 0.04 | 1.34 | 0.06 | **0.05** | 1.22 | 0.02 | 1.32 | 0.05 | 0.07 |
| TOTAL MUFA | 18.19 | 0.21 | 18.78 | 0.29 | 0.18 | 19.10 | 0.13 | 19.57 | 0.28 | 0.17 |
| Linoleic acid (C18:2) | 14.00 | 0.27 | 12.39 | 0.37 | **0.01** | 14.22 | 0.12 | 13.12 | 0.27 | **<0.01** |
| DGLA (C20:3) | 2.35 | 0.07 | 2.07 | 0.09 | **0.04** | 2.05 | 0.04 | 1.81 | 0.08 | **0.02** |
| ARA (C20:4) | 19.73 | 0.21 | 19.39 | 0.29 | 0.44 | 18.75 | 0.14 | 18.32 | 0.32 | 0.26 |
| TOTAL ω6 | 36.12 | 0.29 | 33.84 | 0.39 | **<0.01** | 35.02 | 0.16 | 33.27 | 0.35 | **<0.01** |
| EPA (C20:5) | 0.49 | 0.04 | 0.63 | 0.06 | 0.10 | 0.59 | 0.02 | 0.65 | 0.05 | 0.31 |
| DHA (C22:6) | 4.52 | 0.19 | 5.84 | 0.26 | **<0.01** | 4.93 | 0.10 | 5.83 | 0.23 | **<0.01** |
| TOTAL ω3 | 5.01 | 0.21 | 6.47 | 0.29 | **<0.01** | 5.52 | 0.11 | 6.48 | 0.26 | **<0.01** |
| TOTAL PUFA | 41.12 | 0.24 | 40.31 | 0.32 | 0.10 | 40.54 | 0.15 | 39.88 | 0.33 | 0.09 |
| Trans C18:1 | 0.08 | 0.01 | 0.07 | 0.01 | 0.65 | 0.08 | 0.01 | 0.10 | 0.02 | 0.34 |
| Trans C20:4 | 0.07 | 0.01 | 0.08 | 0.01 | 0.59 | 0.07 | 0.01 | 0.10 | 0.02 | 0.14 |
| TOTAL TRANS | 0.15 | 0.01 | 0.15 | 0.02 | 0.98 | 0.16 | 0.01 | 0.14 | 0.02 | 0.46 |
| **Indexes** | | | | | | | | | | |
| ω6/ω3 | 7.55 | 0.27 | 5.51 | 0.37 | **<0.01** | 6.66 | 0.15 | 5.11 | 0.34 | **<0.01** |
| SFA/MUFA | 2.24 | 0.03 | 2.19 | 0.04 | 0.37 | 2.12 | 0.02 | 2.07 | 0.04 | 0.32 |
| Omega-3 Index | 5.01 | 0.21 | 6.47 | 0.29 | **<0.01** | 5.52 | 0.11 | 6.48 | 0.26 | **<0.01** |
| ∆6D+ELO 20:3/18:2 ^a^ | 0.17 | 0.006 | 0.17 | 0.008 | 0.96 | 0.14 | 0.003 | 0.14 | 0.006 | - |
| ∆5D 20:4/20:3 | 8.60 | 0.31 | 9.51 | 0.41 | 0.15 | 9.45 | 0.21 | 10.55 | 0.47 | 0.05 |
| ∆9D 16:1/16:0 | 0.02 | 0.001 | 0.016 | 0.001 | 0.06 | 0.018 | 0.001 | 0.02 | 0.001 | - |
| ∆9D 18:1/18:0 | 0.91 | 0.02 | 0.98 | 0.02 | 0.07 | 0.99 | 0.01 | 1.00 | 0.01 | 0.67 |
| DNL Index 16:0/18:2 | 1.62 | 0.03 | 1.85 | 0.05 | **<0.01** | 1.59 | 0.02 | 1.72 | 0.03 | **<0.01** |
| PUFA BALANCE | 12.13 | 0.52 | 16.09 | 0.70 | **<0.01** | 13.61 | 0.27 | 16.21 | 0.61 | **<0.01** |
| Peroxidation Index | 136.77 | 1.42 | 145.07 | 1.91 | **0.01** | 136.81 | 0.82 | 141.31 | 1.89 | **0.04** |
| Unsaturation index | 161.53 | 0.92 | 165.51 | 1.24 | **0.04** | 161.25 | 0.61 | 163.57 | 1.37 | 0.15 |

Data is presented as mean ± standard error (SE).

^*^Adjusted for age, sex, and dietary components, extracted from the principal component analysis of dietary nutrient intake (individual FAs, families (SFA, MUFA, and PUFA), total lipids, carbohydrates, and proteins). Post hoc tests were conducted with a Bonferroni adjustment.

^a^ Levene’s test of homogeneity of variance was not met.

- 1. ***Blood biochemistry parameters***

Blood biochemical parameters were determined in a subsample of the study (Table 2). Glucose levels were significantly higher in the adult populations with obesity (p<0.001) but not for the groups with normal weight (p=0.38). Alanine Aminotransferase (ALT/GPT) values were lower for children with obesity compared with adults with obesity (p=0.03) but no differences were observed between groups with normal weight. Cholesterol and triglycerides were statistically higher for adults with obesity (p<0.01 for both) compared to children with obesity, but no differences between groups with normal weight were observed.

**Table 2.** Biochemical values measured in plasma in a fraction of the observed groups.

|  | **Group** **with obesity** | |  | **Group with normal weight** | |  |
| --- | --- | --- | --- | --- | --- | --- |
|  | **Pediatric n=69** | **Adult n=44** | p***** | **Pediatric n=34** | **Adult n=30** | p***** |
|  | **Med (Q1 - Q3)** | **Med (Q1 - Q3)** |  | **Med (Q1 - Q3)** | **Med (Q1 - Q3)** |  |
| Glucose (mg/dL) | 85 (79 - 89.25) | 97 (90.5 - 107.5) | **<0.01** | 84 (81 - 89) | 85 (79.75 - 92) | 0.38 |
| Uric Acid (mg/dL) | 4.95 (4.375 - 5.7) | 5.6 (4.9 - 6.95) | **<0.01** | 3.95 (3.37 - 4.62) | 4.75 (3.8 - 5.22) | **0.03** |
| Total Cholesterol (mg/dL) | 150 (132.7 - 172) | 180 (158 - 211) | **<0.01** | 165 (148.5 - 186.7) | 176.5 (141.7 - 206.2) | 0.46 |
| Triglycerides (mg/dL) | 76 (55.5 - 108.7) | 123 (89.5 - 180.5) | **<0.01** | 65.5 (46 - 86) | 68 (58.75 - 84.75) | 0.48 |
| HDL cholesterol (mg/dL) | 44.6 (40.0 - 54.25) | 47 (41.75 - 56) | 0.32 | 55 (48.5 - 64.5) | 59 (48.5 - 71) | 0.48 |
| LDL cholesterol (mg/dL) | 88.4 (71.25 - 98) | 118 (95 - 141) | **<0.01** | 95 (77 - 110) | 102 (72.5 - 121) | 0.35 |
| AST/GOT (U/L) | 22 (19 - 26.25) | 20 (16.5 - 26.5) | 0.12 | 26 (22 - 27) | 19 (16.75 - 23.5) | **<0.01** |
| ALT/GPT (U/L) | 18.5 (15 - 23.25) | 23 (15 - 35.5) | **0.03** | 16 (13.75 - 18) | 17 (12.75 - 21.25) | 0.59 |
| Bilirubin (mg/dL) | 0.4 (0.3 - 0.6) | 0.4 (0.2 - 0.5) | 0.5 | 0.6 (0.4 - 1) | 0.4 (0 - 0.625) | **0.01** |

Data expressed as medians and quartile 1 and quartile 3 (Med (Q1 - Q3)).

* Not normally distributed variables. A Mann-Whitney U test was carried out.

- 1. ***Food Groups***

In order to compare the dietary pattern of each study group, we considered food groups as shown in **Table S1** (supplementary material) and observed that both adults with obesity and with nomal weight showed a higher intake of vegetables, olive oil, white meat, oily fish, sugary drinks and dried fruits and nuts (p<0.01 for all) compared to group of children. On the other hand, cereals, legumes and juice intakes were higher for the pediatric population (p<0.01 for both obese and normoweight). In any case, these results should be taken into account from the perspective that, adults, reported a higher intake of daily calories, so when comparing food groups in grams per day units, it is normal to observe differences.

- 1. ***Nutrient intake***

Regarding macro-nutrient intake shown in **Table 3**, as both population groups differ in terms of quantity requirements, to compare each other, variables were expressed in % of energy obtained from each macro-micronutrient. Differences between obese adults and children were observed for total calories (Kcal/day) and the intake of carbohydrates, simple sugars and total lipids. Some of these differences, such as calories differences (p<0.01) can be related to different requirements depending on age. Distribution of the energy intake obtained from macronutrients differs between both populations for carbohydrates (p<0.01), being higher for pediatric population. Although adults showed, proportionally, a higher intake of total lipids than children (p<0.01), the pediatric population showed higher intake of stearic acid (p=0.04). Oleic acid and total MUFA intake were higher for the adult population. Regarding PUFAs, total ω-6, corresponding ω-6 fatty acids (LA and AA) and total ω-3 dietary intake were higher for adults than for children. The ω-6/ω-3 ratio was lower for the pediatric population (p<0.01).

**Table 3.** Macronutrients and individual fatty acids (FA) intake expressed as % energy (%*E*) in pediatric and adult groups with obesity.

|  | **Pediatric group with obesity, n=83** | | **Adult group with obesity, n=61** | | **Mann-Whitney U test** |
| --- | --- | --- | --- | --- | --- |
|  | Mean | SD | Mean | SD | p |
| **Macronutrient** | | | | | |
| Calories (Kcal/day) | 2044.1 | 564.3 | 2480.1 | 794.2 | **<0.01** |
| Proteins (%E) | 16.5 | 2.1 | 16.3 | 3.2 | 0.54 |
| Carbohydrates (%E) | 46.7 | 5.3 | 36.3 | 6.6 | **<0.01*** |
| Simple sugars (%E) | 21.7 | 4.9 | 19.1 | 6.4 | **<0.01** |
| Lipids (%E) | 33.6 | 6.3 | 42.6 | 6.3 | **<0.01*** |
| **Individual fatty acids** | | | | | |
| C14:0 | 1.0 | 0.5 | 0.8 | 0.3 | 0.08 |
| C16:0 | 6.1 | 1.3 | 5.6 | 1.0 | **0.02*** |
| C18:0 | 2.3 | 0.6 | 2.3 | 0.5 | 0.93 |
| Total SFA | 10.8 | 2.9 | 11.2 | 2.2 | 0.09 |
| C16:1 | 0.5 | 0.1 | 0.5 | 0.1 | 0.86 |
| C18:1 | 14.1 | 3.5 | 18.9 | 4.1 | **<0.01*** |
| Total MUFA | 15.0 | 3.6 | 19.9 | 4.2 | **<0.01*** |
| C18:2 | 4.1 | 1.7 | 6.9 | 2.5 | **<0.01** |
| C20:4 | 0.04 | 0.01 | 0.08 | 0.04 | **<0.01** |
| Total ω6 | 4.1 | 1.7 | 7.0 | 2.5 | **<0.01** |
| C18:3 | 0.54 | 0.13 | 0.79 | 0.34 | **<0.01** |
| C20:5 (EPA) | 0.07 | 0.06 | 0.07 | 0.05 | 0.24 |
| C22:5 (DPA) | 0.02 | 0.01 | 0.02 | 0.01 | 0.48 |
| 22:6 (DHA) | 0.15 | 0.09 | 0.14 | 0.08 | 0.62 |
| Total ω3 | 0.79 | 0.22 | 1.03 | 0.39 | **<0.01** |
| Total PUFA | 5.1 | 1.8 | 8.2 | 2.7 | **<0.01** |
| ω6/ω3 | 5.5 | 2.2 | 7.2 | 2.8 | **<0.01** |

Data presented as mean and standard deviation (SD). Not normally distributed variables. A Mann-Whitney U test was carried out. *Normally distributed variables, an independent-samples t-test was performed.

- 1. ***RBC FA-s and blood biochemical parameters correlation***

Different correlation profiles between RBC FAs and biochemical parameters were observed for children and adults with obesity (see heatmaps in Supplementary **Figure S1** and Supplementary **Figure S2**, respectively). For the pediatric population inverse correlations between LA and total cholesterol and LDL cholesterol were observed, as well as an inverse correlation between EPA and triglycerides. For adults, other correlations were observed. DHA correlated positively with AST and ALT. At the same time, oleic acid showed an inverse correlation with LDL cholesterol and stearic acid correlated inversely with HDL cholesterol.

- 1. ***RBC FAs and food groups values correlation***

Different correlations between RBC FA-s and food groups were observed for adults and children with obesity (see heatmaps in supplementary **Figure S3** and **Figure S4**, respectively). Children showed a positive correlation between EPA in RBC and white fish intake, and DHA in RBC with oily fish. Read meat correlated positively with cis-vaccenic acid in RBC. For adults, only positive correlations between trans fatty acids in RBC and eggs was observed.

1. **Discussion**

To our knowledge this is the first time that a comparison of RBC membrane FA composition between adults and children with obesity has been made to determine metabolic differences, in order to establish dietary requirements that can contribute to design more precise nutritional strategies based on dietary fat quality to manage obesity at different age stages. The fact that the RBC membrane fatty acid composition is close to that of hepatocytes, having saturated (43% vs 42%), monounsaturated (23.0% vs 23.8%), polyunsaturated ω-6 (27.6 vs 27.4%) and ω-3 (5.7% vs 4.6%) fatty acid residues in almost similar quantities([31](#_ENREF_31)), is an important observation, since the RBC examination avoids to run invasive investigations, especially in children.

Regarding individual nutrient intake, differences were observed between children and adults with obesity, taking into account that the nutritional recommendations for both population groups differ because metabolism requirements are different([32](#_ENREF_32)). Two main reasons to measure dietary intake through a FFQ questionnaire were: to establish the eating pattern of each group and to consider dietary intake as a confounding factor in the Ancova analysis, of the study of metabolic differences, between the children and adults with obesity ([25](#_ENREF_25)). The elimination of the variability generated by diet in the RBC FA profile, allows us to focus on the metabolic differences between adults and children with obesity. Concerning the dietary pattern of each group, the higher levels of ω6/ω3 ratio in RBCs for adults with obesity was an interesting result, showing its relationship with the quality and not with the quantity of lipids intake.

Measurement of RBC FAs revealed two differentiated profiles between children and adults with obesity, where not all the differences were attributable to age, as those results have been compared with a population of adults and children with normal weight, and can be due to metabolic differences.

Related with age, differences in PUFA levels between pediatric and adult, for both group with normal weight and obesity, were observed. Adults with obesity showed a proportionally, higher intake of ω6 FAs than children with obesity, contributing to a higher intake ratio of ω6/ω3, but in the RBC membrane profile, adults with obesity showed lower levels of ω6/ω3 FA ratio. Even if the children´s intake of ω6 was lower compared with adults, a higher value of ω6/ω3 FA ratio in RBC membrane was determined. It can be observed, that for children the contribution to ω6 levels is given, in a significantly higher manner, by linoleic and DGLA acids, whereas for ω3 levels, is given by DHA, revealing a different metabolic fate of the dietary intakes. Certainly, the greater proportion of DHA needed for heart and brain tissues ([33](#_ENREF_33)), could be responsible for a higher distribution of this FA in children compared to the adult group, because of the growth associated with that stage of life([34](#_ENREF_34)). In our opinion, levels of DHA specifically determined in cell membranes, in particular in mature RBC membranes where PUFA ω-3 were found higher than in non-selected RBC([18](#_ENREF_18)), should be considered as important information of the bioavailability of essential or semi-essential FA for the fundamental building up of the membrane compartment. Formation of membranes is needed for living organisms([35](#_ENREF_35)) and must be combined with an appropriate composition of the FA pool to avoid critical unbalances. The fatty acid-based membrane lipidomics is a diagnostic tool that provides an important piece of information in the puzzle of the metabolic pathways of health and disease([18](#_ENREF_18)).

Similar connections with mediator formation can be inferred for the ω6 FAs in RBC, as LA and DGLA levels were higher for the pediatric population compared with the adult population, indistinctly for groups with obesity and normal weight. Higher values of DGLA in children compared to adults can indicate a metabolic connection with mediators for inflammatory, immune and defense processes, since this FA is a precursor of series 1 prostaglandins, like PGE1, connected with the cAMP activity and this can occur in a higher rate in children, regardless of the intake([36](#_ENREF_36)).

These metabolic differences appear as important factors to evaluate the fat quality and quantity especially in diseases which involve fats, such as obesity. The adequate nutritional strategies for each population group should be personalized, for example, reinforcing the ω-3 FA-s intake recommendation in the pediatric population compared to the adult population, because of their higher requirements([8](#_ENREF_8)). Increasing dietary intake of ω3 sources such as oily fish and seafood, especially cold-water fatty fish (sardine, tuna, salmon, mackerel) and vegetable sources such as walnuts, chia seeds, flaxseed, or even with a personalized nutraceutical plan ([37](#_ENREF_37)) could be a roadmap.

When comparing MUFA and SFA-s levels in RBC, adults with obesity showed higher levels of palmitic acid and cis-vaccenic acid compared with children with obesity, while these differences were not observed among the normoweight groups (Table 1). The % of energy obtained from palmitic acid in the diet, appears in a higher proportion for children with obesity than for adults with obesity, but contrary than expected, adults with obesity, reflect a higher value of RBC palmitic acid in the membrane profile.

The higher value in the DNL index in adults with obesity compared with children with obesity, reflects a higher *de novo* synthesis of lipids, that can explain the higher levels of palmitic acid in adults with obesity([38](#_ENREF_38)). Together with this higher DNL index, a tendency of a reduced activity of D9D (p=0.06) for adults with obesity compared with children with obesity can be observed. This reduced activity of the enzyme is correlated to factors that have been recalled several times in the SFA pathway, as: the absence of enzymatic cofactors, the inhibition of desaturase activity and liver impairment.([39](#_ENREF_39)) A high‐carbohydrate diet can increase rates of DNL, that has been suggested to contribute to the pathogenesis of non‐alcoholic fatty liver disease (NAFLD), linked in its turn to the development of type 2 diabetes mellitus.([40](#_ENREF_40))

As desaturase transformation prevents SFA accumulation and toxicity triggering hepatocellular apoptosis and liver damage([41](#_ENREF_41)), adults with obesity, that have suffered the accumulation of SFA for years, or at least for a longer time than children, might be a plausible reason that D9D presents a tendency of less activity in adults than in children with obesity.

In any case, recommendations should consider these differences between adults and children, highlighting the importance of not promoting *de novo* synthesis in adults by lowering the intake of SFA and simple carbohydrates. Moreover, increasing the intake of PUFAs has been associated to inhibitory effects on SFA and MUFA biosynthesis([42](#_ENREF_42)) and could be seen as a proper recommendation.

According to biochemical parameters, even if most of them are within the optimal ranges in both groups with obesity and normal weight, it is remarkable that glucose, total cholesterol, LDL cholesterol, triglycerides and ALT/GPT showed significative lower values for children with obesity compared with adults with obesity, fact that was not observed in the group with normal weight. Additionally, differentiated correlations between biochemical parameters and RBC FAs were observed. For children, higher levels of LA inversely correlated with LDL cholesterol and total cholesterol were determined (Supplementary Figure 1). This correlation has been previously reported also for both, circulating LA and RBC LA and cholesterol([43](#_ENREF_43), [44](#_ENREF_44)). Food groups with higher content of LA, such as nuts, would be recommendable to lower LDL cholesterol levels in pediatric populations. On the other hand, adults showed an inverse correlation with oleic acid and LDL cholesterol, so higher consumption of food groups containing this FA-s, such as olive oil, would be recommendable to reduce LDL cholesterol levels. SFA/MUFA ratio in adults, showed a positive correlation with LDL cholesterol, so the replacement of SFA with MUFAs in dietary intake would be advisable in order to reduce LDL cholesterol levels. For adults, DHA and total ω3 showed positive correlations with ALT and AST. Anyway, it has been reported in the literature that PUFAs can only decrease ALT and AST after long term supplementation in children ([45](#_ENREF_45)).

As a limitation of the study, the uneven group distribution, the number of each group, should be noted even if in this type of observational studies, a perfect match is hard to achieve. At the same time, the indirect measurement of enzyme activity by the ratio between product and precursors, although very popular, could be considered as a limitation of the study and should be measured directly to emphasize and reaffirm the conclusions obtained. Another limitation of the study was the use of a population subgroup for the analysis of differences in biochemical parameters, which reduces the possibility of finding more significant differences and correlations.

In conclusion, the present study establishes the differences of RBC FA profiles, between children and adult with obesity, demonstrating that both groups have differentiated profiles. Children with obesity present higher LA, DGLA and total ω6 values, along with lower DHA and total ω3 values, compared to adults with obesity, even after adjusting the values by their dietary intakes. At the same time, children with obesity presented lower levels of palmitic acid and a lower value of the *de novo lipogenesis* index compared to adults with obesity. These differences must be considered to provide more specific food group recommendations based on individual FA needs, rather than giving general recommendations for population with obesity, as a whole and regardless of age.

**Abbreviations AA:** arachidonic acid; ANCOVA: analysis of covariance; BMI: body mass index; DGLA: Dihomo-gamma-linolenic acid; DHA: docosahexaenoic acid; DPA: docosapentaenoic acid; EDTA: ethylenediaminetetraacetic acid; EFA: essential fatty acid; EPA: eicosapentaenoic acid; FA: fatty acid; FFQ: food frequency questionnaire; FAME: fatty acid methyl ester; KMO: Kaiser-Meyer-Olkin; MeOH: methyl alcohol; MUFA: monounsaturated fatty acids; KOH: potassium hydroxide; LA: linoleic acid; PUFA: polyunsaturated fatty acids; RBC: red blood cell; SFA: saturated fatty acids; SCD1: stearoyl-CoA desaturase-1; SD: standard deviation; TFA: trans fatty acids; UI: unsaturation index; PI: peroxidation index; PCA: principal component analysis.

**Author contributions:** Conceptualization, S.A. and I.J.; Methodology, S.A., I.R., S.G., L.C., C.F.; Formal Analysis, I.J., S.G., I.R., N.T., G.G., O.V., A.V.L; Investigation, I.J., S.A.; Data Curation, I.J., K.P.; Writing – Original Draft Preparation, I.J., S.A.; Writing – Review & Editing, I.J., S.A., I.T., C.F.; Supervision, S.A, C.F. All authors have read and approved the final manuscript.

**Acknowledgments:** IJ thanks the Department of Economic Development and Infrastructures of the Basque Government for receiving a PhD grant for young researchers in the scientific-technological and business environment of the Basque agricultural and food sector. This paper is contribution nº xxx from AZTI, Food Research, Basque Research and Technology Alliance (BRTA).

**Funding:** This work was supported by the Department of Environment: Territorial Planning: Agriculture and Fisheries of the Basque Country Government (EMOI project funding by ELKARTEK 2017; Innovation Fund 2017); the Department of Health of the Basque Government (2017222033: OBESIA 2016-2019); the Centre for the Development of Industrial Technology (CDTI) of the Spanish Ministry of Science and Innovation under the grant agreement: TECNOMIFOOD project (CER-20191010); the INC (INTERNATIONAL NUT AND DRIED FRUIT COUNCIL) under the grant agreement OBINUT project (2016(II)-R01) and a fellowship from Fundación Gangoiti 2018/19 y 2019/2020.

**Conflicts of Interest:** AVL and FDN receive their salaries from Lipinutragen. CF is Scientific Director at Lipinutragen, born as spin-off company of the Consiglio Nazionale delle Ricerche in Bologna (Italy).

**Ethics approval and consent to participate:** The study protocol was approved by the Euskadi Clinical Research Ethics Committee (permission number PI2016181) and investigations were carried out following the rules of the Declaration of Helsinki of 1975 (https://www.wma.net/what-we-do/medical-ethics/declaration-of-helsinki/), revised in 2013. Subjects under study were included after acceptance (of the parents) to participate in the study and signing of informed consent.

1. **References**

1. Organization WH. Obesity and overweight. 2016; Available from: https://[www.who.int/en/news-room/fact-sheets/detail/obesity-and-overweight](http://www.who.int/en/news-room/fact-sheets/detail/obesity-and-overweight).

2. Tobias DK, Chen M, Manson JE, Ludwig DS, Willett W, Hu FB. Effect of low-fat diet interventions versus other diet interventions on long-term weight change in adults: a systematic review and meta-analysis. Lancet Diabetes Endocrinol. 2015;3(12):968-79.

3. Mensink RP, Zock PL, Kester AD, Katan MB. Effects of dietary fatty acids and carbohydrates on the ratio of serum total to HDL cholesterol and on serum lipids and apolipoproteins: a meta-analysis of 60 controlled trials. The American journal of clinical nutrition. 2003;77(5):1146-55.

4. Sacks FM, Katan M. Randomized clinical trials on the effects of dietary fat and carbohydrate on plasma lipoproteins and cardiovascular disease. Am J Med. 2002;30(113):00987-1.

5. Jakobsen MU, O'Reilly EJ, Heitmann BL, Pereira MA, Bälter K, Fraser GE, et al. Major types of dietary fat and risk of coronary heart disease: a pooled analysis of 11 cohort studies. The American journal of clinical nutrition. 2009;89(5):1425-32.

6. Koliaki C, Spinos T, Spinou Μ, Brinia Μ-E, Mitsopoulou D, Katsilambros N. Defining the Optimal Dietary Approach for Safe, Effective and Sustainable Weight Loss in Overweight and Obese Adults. Healthcare (Basel). 2018;6(3):73.

7. Teixeira PJ, Carraça EV, Marques MM, Rutter H, Oppert JM, De Bourdeaudhuij I, et al. Successful behavior change in obesity interventions in adults: a systematic review of self-regulation mediators. BMC Med. 2015;13(84):015-0323.

8. EFSA Panel on Dietetic Products N, Allergies. Scientific Opinion on Dietary Reference Values for fats, including saturated fatty acids, polyunsaturated fatty acids, monounsaturated fatty acids, trans fatty acids, and cholesterol. EFSA Journal. 2010;8(3):1461.

9. Fao. Fats and fatty acids in human nutrition: Report of an expert consultation2010.

10. Gow ML, Ho M, Burrows TL, Baur LA, Stewart L, Hutchesson MJ, et al. Impact of dietary macronutrient distribution on BMI and cardiometabolic outcomes in overweight and obese children and adolescents: a systematic review. Nutr Rev. 2014;72(7):453-70.

11. Camilleri M, Staiano A. Insights on Obesity in Children and Adults: Individualizing Management. Trends in Endocrinology & Metabolism. 2019;30(10):724-34.

12. Abbott SK, Else PL, Atkins TA, Hulbert AJ. Fatty acid composition of membrane bilayers: Importance of diet polyunsaturated fat balance. Biochimica et Biophysica Acta (BBA) - Biomembranes. 2012;1818(5):1309-17.

13. Perona JS. Membrane lipid alterations in the metabolic syndrome and the role of dietary oils. Biochimica et Biophysica Acta (BBA) - Biomembranes. 2017;1859(9, Part B):1690-703.

14. Escribá PV. Membrane-lipid therapy: A historical perspective of membrane-targeted therapies — From lipid bilayer structure to the pathophysiological regulation of cells. Biochimica et Biophysica Acta (BBA) - Biomembranes. 2017;1859(9, Part B):1493-506.

15. Ferreri C, Chatgilialoglu C. Role of fatty acid-based functional lipidomics in the development of molecular diagnostic tools. Expert Rev Mol Diagn. 2012;12(7):767-80.

16. Genio G. Morbid Obesity is Associated to Altered Fatty Acid Profile of Erythrocyte Membranes. Journal of Diabetes & Metabolism. 2015;06.

17. Ghezzo A, Visconti P, Abruzzo PM, Bolotta A, Ferreri C, Gobbi G, et al. Oxidative Stress and Erythrocyte Membrane Alterations in Children with Autism: Correlation with Clinical Features. PloS one. 2013;8(6).

18. Ferreri C, Masi A, Sansone A, Giacometti G, Larocca AV, Menounou G, et al. Fatty Acids in Membranes as Homeostatic, Metabolic and Nutritional Biomarkers: Recent Advancements in Analytics and Diagnostics. Diagnostics. 2016;7(1).

19. Sánchez González E, Carrascosa Lezcano A, Fernández García JM, Ferrández Longás A, López de Lara D, López-Siguero JP. Estudios españoles de crecimiento: situación actual, utilidad y recomendaciones de uso. Anales de Pediatría. 2011;74(3):193.e1-.e16.

20. Carrascosa A, Fernández JM, Fernández C, Ferrández A, López-Siguero JP, Sánchez E, et al. Estudios españoles de crecimiento 2008. Nuevos patrones antropométricos. Endocrinología y Nutrición. 2008;55(10):484-506.

21. Madrigal C, Soto-Mendez MJ, Hernandez-Ruiz A, Ruiz E, Valero T, Avila JM, et al. Dietary and Lifestyle Patterns in the Spanish Pediatric Population (One to <10 Years Old): Design, Protocol, and Methodology of the EsNuPI Study. Nutrients. 2019;11(12).

22. Fernandez-Ballart JD, Pinol JL, Zazpe I, Corella D, Carrasco P, Toledo E, et al. Relative validity of a semi-quantitative food-frequency questionnaire in an elderly Mediterranean population of Spain. Br J Nutr. 2010;103(12):1808-16.

23. Sansone A, Tolika E, Louka M, Sunda V, Deplano S, Melchiorre M, et al. Hexadecenoic Fatty Acid Isomers in Human Blood Lipids and Their Relevance for the Interpretation of Lipidomic Profiles. PloS one. 2016;11(4).

24. Giacometti G, Ferreri C, Sansone A, Chatgilialoglu C, Marzetti C, Spyratou E, et al. High predictive values of RBC membrane-based diagnostics by biophotonics in an integrated approach for Autism Spectrum Disorders. Scientific reports. 2017;7(1):017-10361.

25. Amezaga J, Arranz S, Urruticoechea A, Ugartemendia G, Larraioz A, Louka M, et al. Altered Red Blood Cell Membrane Fatty Acid Profile in Cancer Patients. Nutrients. 2018;10(12).

26. Van Der Vegt SGL, Ruben AMT, Werre JM, Palsma DMH, Verhoef CW, de Gier J, et al. Counterflow centrifugation of red cell populations: a cell age related separation technique. British Journal of Haematology. 1985;61(3):393-403.

27. Breil C, Abert Vian M, Zemb T, Kunz W, Chemat F. "Bligh and Dyer" and Folch Methods for Solid-Liquid-Liquid Extraction of Lipids from Microorganisms. Comprehension of Solvatation Mechanisms and towards Substitution with Alternative Solvents. International journal of molecular sciences. 2017;18(4).

28. Ferreri C, Faraone Mennella MR, Formisano C, Landi L, Chatgilialoglu C. Arachidonate geometrical isomers generated by thiyl radicals: the relationship with trans lipids detected in biological samples. Free Radic Biol Med. 2002;33(11):1516-26.

29. Jacobs S, Jäger S, Jansen E, Peter A, Stefan N, Boeing H, et al. Associations of Erythrocyte Fatty Acids in the De Novo Lipogenesis Pathway with Proxies of Liver Fat Accumulation in the EPIC-Potsdam Study. PloS one. 2015;10(5).

30. Jauregibeitia IP, K.; Rica, I.; Tueros, I.; Velasco, O.; Grau, G.; Trebolazabala, N.; Castaño, L.; Larocca, A.V.; Ferreri, C.; Arranz, S. Fatty Acid Profile of Mature Red Blood Cell Membranes and Dietary Intake as a New Approach to Characterize Children with Overweight and Obesity. Nutrients. 2020;12.

31. Lauritzen L, Hansen HS, Jørgensen MH, Michaelsen KF. The essentiality of long chain n-3 fatty acids in relation to development and function of the brain and retina. Prog Lipid Res. 2001;40(1-2):1-94.

32. Khan FA, Fisher JG, Sparks EA, Jaksic T. Metabolism of Infants and Children. In: Puri P, editor. Pediatric Surgery: General Principles and Newborn Surgery. Berlin, Heidelberg: Springer Berlin Heidelberg; 2020. p. 231-43.

33. Harayama T, Riezman H. Understanding the diversity of membrane lipid composition. Nat Rev Mol Cell Biol. 2018;19(5):281-96.

34. Weiser MJ, Butt CM, Mohajeri MH. Docosahexaenoic Acid and Cognition throughout the Lifespan. Nutrients. 2016;8(2):99-.

35. Sender R, Fuchs S, Milo R. Revised Estimates for the Number of Human and Bacteria Cells in the Body. PLoS Biol. 2016;14(8).

36. Kirtland SJ. Prostaglandin E1: A review. Prostaglandins, Leukotrienes and Essential Fatty Acids (PLEFA). 1988;32(3):165-74.

37. Jauregibeitia I, Portune K, Amezaga J, Tueros I, Arranz S. A Journey through ?-3 Supplements: Future Perspectives for Precision Nutrition. Journal of Food and Nutrition Research. 2020;8(10):556-60.

38. Alves-Bezerra M, Cohen DE. Triglyceride Metabolism in the Liver. Compr Physiol. 2017;8(1):1-8.

39. Ferreri C, Chatgilialoglu, C. Membrane Lipidomics for Personalized Health. Chichester, UK: John Wiley & Sons; 2015.

40. Sanders FW, Griffin JL. De novo lipogenesis in the liver in health and disease: more than just a shunting yard for glucose. Biol Rev Camb Philos Soc. 2016;91(2):452-68.

41. Silbernagel G, Kovarova M, Cegan A, Machann J, Schick F, Lehmann R, et al. High Hepatic SCD1 Activity Is Associated with Low Liver Fat Content in Healthy Subjects under a Lipogenic Diet. The Journal of Clinical Endocrinology & Metabolism. 2012;97(12):E2288-E92.

42. Albracht-Schulte K, Kalupahana NS, Ramalingam L, Wang S, Rahman SM, Robert-McComb J, et al. Omega-3 fatty acids in obesity and metabolic syndrome: a mechanistic update. The Journal of nutritional biochemistry. 2018;58:1-16.

43. Burns JL, Nakamura MT, Ma DWL. Differentiating the biological effects of linoleic acid from arachidonic acid in health and disease. Prostaglandins, Leukotrienes and Essential Fatty Acids. 2018;135:1-4.

44. Ruiz-Núñez B, Kuipers RS, Luxwolda MF, De Graaf DJ, Breeuwsma BB, Dijck-Brouwer DA, et al. Saturated fatty acid (SFA) status and SFA intake exhibit different relations with serum total cholesterol and lipoprotein cholesterol: a mechanistic explanation centered around lifestyle-induced low-grade inflammation. The Journal of nutritional biochemistry. 2014;25(3):304-12.

45. Chen LH, Wang YF, Xu QH, Chen SS. Omega-3 fatty acids as a treatment for non-alcoholic fatty liver disease in children: A systematic review and meta-analysis of randomized controlled trials. Clin Nutr. 2018;37(2):516-21.
