# Supplementary material for: Molecular Differences Based on Erythrocyte Fatty Acid Profile to Personalize Dietary Strategies between Adults and Children with Obesity
Source: Metabolites. 2021 Jan 8;11(1):43. doi: 10.3390/metabo11010043 (PMC7827034; doi:10.3390/metabo11010043)
Supplement: Supplementary file 1 [file metabolites-11-00043-s001.zip › Suplementary Figure 2_Heatplot_correlation_Adults_biochem vs RBC FA_.pdf]

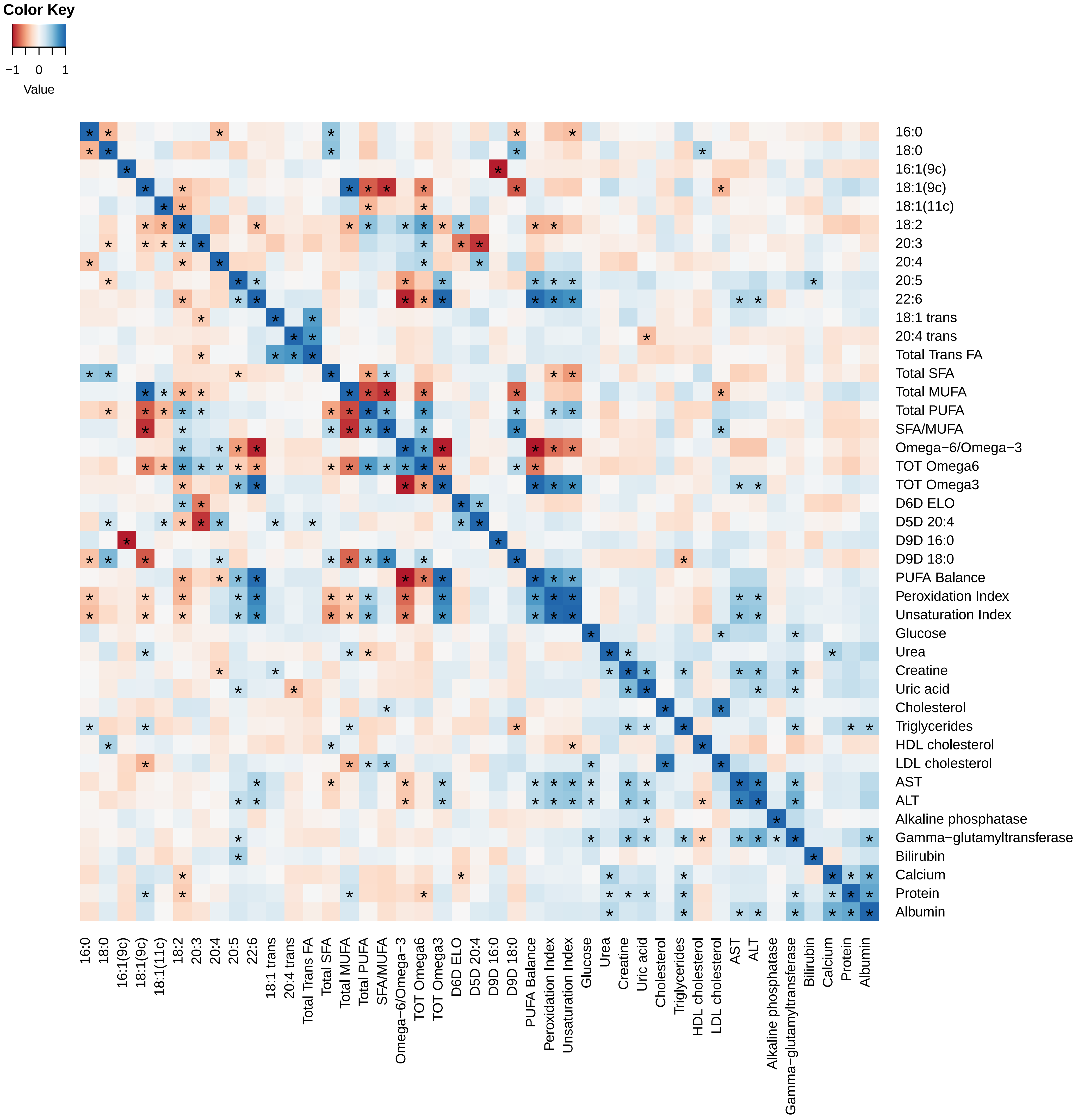
Color Key
−1 0 1
Value
16:0
18:0
16:1(9c)
18:1(9c)
18:1(11c)
18:2
20:3
20:4
20:5
22:6
18:1 trans
20:4 trans
Total Trans FA
Total SFA
Total MUFA
Total PUFA
SFA/MUFA
Omega−6/Omega−3
TOT Omega6
TOT Omega3
D6D ELO
D5D 20:4
D9D 16:0
D9D 18:0
PUFA Balance
Peroxidation Index
Unsaturation Index
Glucose
Urea
Creatine
Uric acid
Cholesterol
Triglycerides
HDL cholesterol
LDL cholesterol
AST
ALT
Alkaline phosphatase
Gamma−glutamyltransferase
Bilirubin
Calcium
Protein
Albumin
