# Supplementary material for: Molecular Differences Based on Erythrocyte Fatty Acid Profile to Personalize Dietary Strategies between Adults and Children with Obesity
Source: Metabolites. 2021 Jan 8;11(1):43. doi: 10.3390/metabo11010043 (PMC7827034; doi:10.3390/metabo11010043)
Supplement: Supplementary file 1 [file metabolites-11-00043-s001.zip › Table S1.docx]

| **Food Groups (g/day)** | | | **Group with obesity** | | | **Group with normal weight** | | |
| --- | --- | --- | --- | --- | --- | --- | --- | --- |
|  | Pediatric | Adult | | p* | Pediatric | | Adult | p* |
|  | Med (Q1 - Q3) | Med (Q1 - Q3) | |  | Med (Q1 - Q3) | | Med (Q1 - Q3) |  |
| Fruits | 390 (264 - 577) | 353 (231 - 682) | | 0.74 | 413 (297 - 531) | | 361 (210 - 691) | 0.72 |
| Vegetables | 134 (76 - 237) | 296 (158 - 442) | | **<0.01** | 160 (100 - 248) | | 313 (222 - 476) | **<0.01** |
| Cereals | 132 (102 - 185) | 98 (60 - 175) | | **<0.01** | 158 (117 - 210) | | 114 (94 - 224) | 0.09 |
| Legumes | 80 (54 - 96) | 43 (32 - 64) | | **<0.01** | 91 (50 - 102) | | 48 (40 - 86) | **<0.01** |
| Olive oil | 15 (12 - 38) | 38 (24 - 39) | | **<0.01** | 15 (15 - 38) | | 20 (16 - 38) | **0.04** |
| Dairy products | 298 (207 - 374) | 268 (163 - 509) | | 0.91 | 325 (254 - 513) | | 306 (175 - 436) | 0.17 |
| Eggs | 15 (15 - 35) | 30 (10 - 30) | | 0.45 | 15 (15 - 35) | | 30 (0 - 30) | 0.21 |
| Red meat | 21 (21 - 50) | 36 (18 - 63) | | 0.13 | 21 (21 - 21) | | 36 (18 - 47) | **<0.01** |
| White meat | 50 (21 - 50) | 98 (54 - 116) | | **<0.01** | 50 (21 - 50) | | 36 (0 - 112) | 0.91 |
| Dried Fruits and nuts | 1 (0 - 3) | 6 (0 - 21) | | **<0.01** | 2 (0 - 6) | | 7 (2 - 36) | **<0.01** |
| Lean fish | 27 (27 - 27) | 21 (11 - 64) | | 0.91 | 27 (27- 27) | | 21 (21 - 21) | **<0.01** |
| Oily fish and shellfish | 27 (13 - 35) | 61 (39 - 108) | | **<0.01** | 27 (13 - 27) | | 68 (47- 126) | **<0.01** |
| Sugary drinks | 16 (0 - 54) | 29 (14 - 86) | | **<0.01** | 18 (0 - 43) | | 14 (0 - 32) | 0.18 |
| Juices | 80 (29 - 243) | 14 (0 - 71) | | **<0.01** | 71 (27 - 250) | | 14 (0 - 89) | **<0.01** |

**Table S1.** Food groups intake

Data expressed as medians and quartile 1 and quartile 3. *Not normally distributed variables. A Mann-Whitney U test was carried out.
