# Supplementary figures and images for: Molecular Differences Based on Erythrocyte Fatty Acid Profile to Personalize Dietary Strategies between Adults and Children with Obesity
Source: Metabolites. 2021 Jan 8;11(1):43. doi: 10.3390/metabo11010043 (PMC7827034; doi:10.3390/metabo11010043)

Color Key

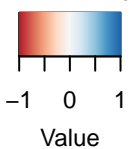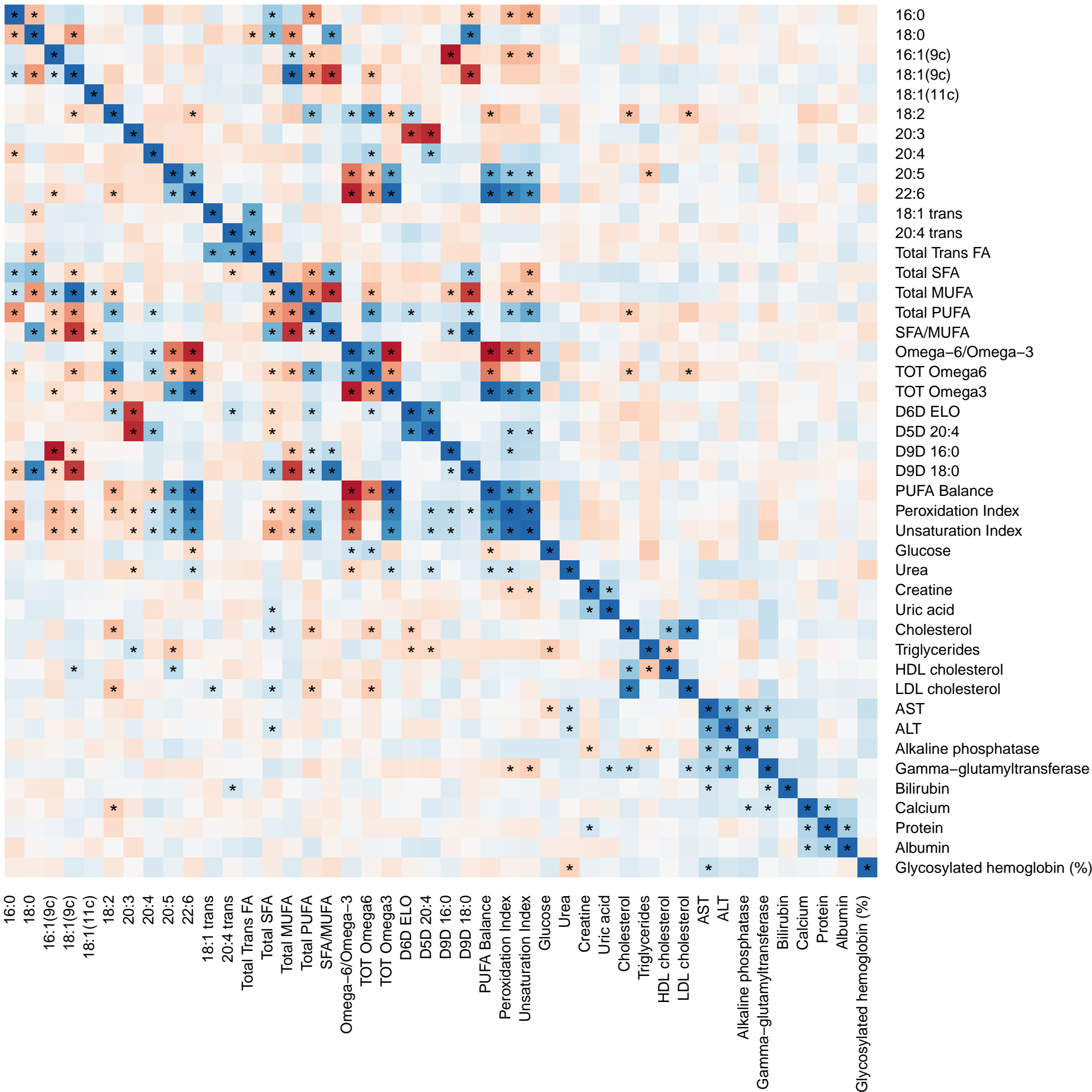

Supplement: Supplementary file 1 [file metabolites-11-00043-s001.zip › Suplementary Figure 1_Heatplot_correlation_children_biochem vs RBC FA_.pdf]

Color Key

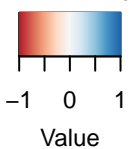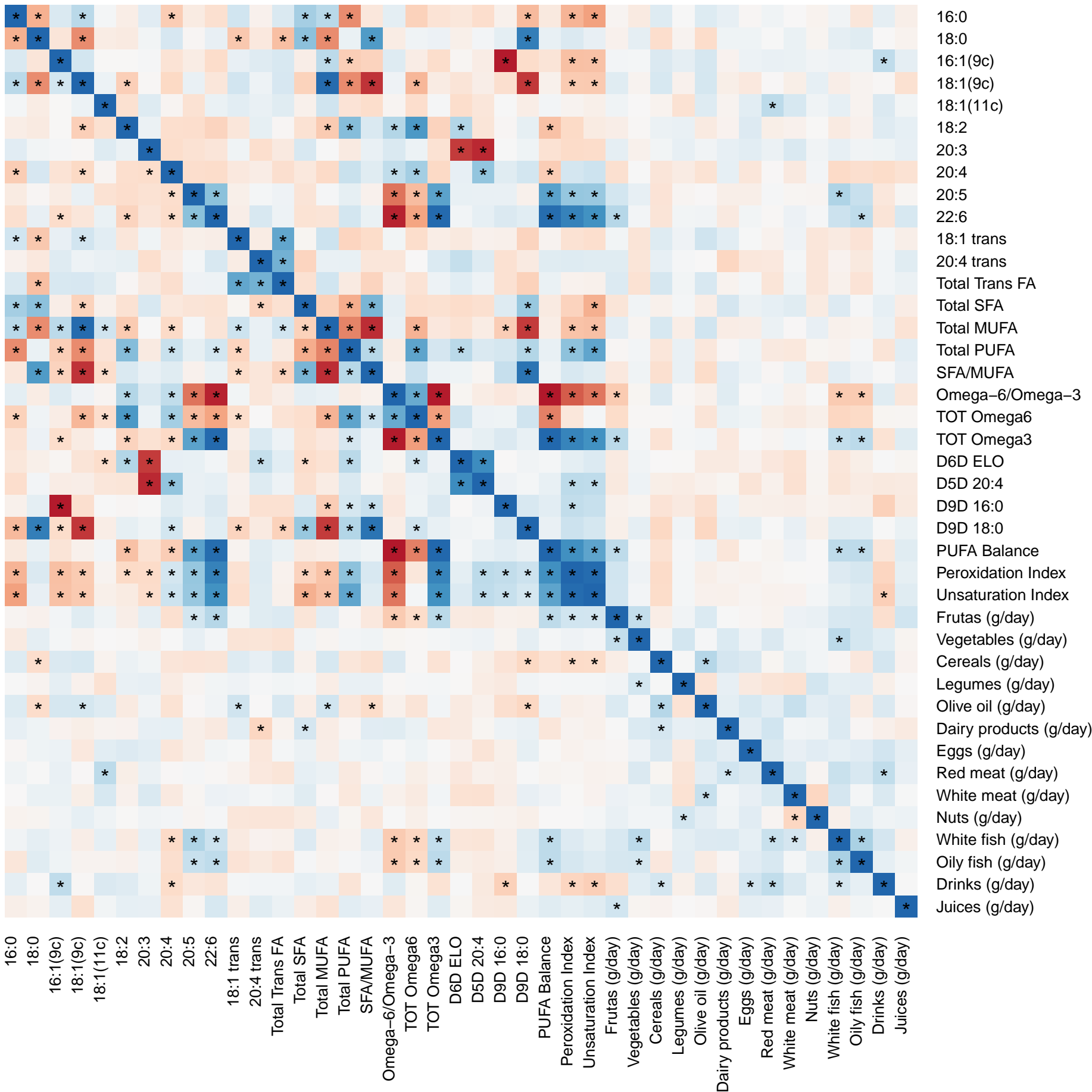

Supplement: Supplementary file 1 [file metabolites-11-00043-s001.zip › Suplementary Figure 3_Heatplot_correlation_children_Food Groups vs RBC FA.pdf]
